# Bronchopulmonary Dysplasia: Comparison Between the Two Most Used Diagnostic Criteria

**DOI:** 10.3389/fped.2018.00397

**Published:** 2018-12-12

**Authors:** Enrique Gomez Pomar, Vanessa A. Concina, Aaron Samide, Philip M. Westgate, Henrietta S. Bada

**Affiliations:** ^1^Division of Neonatology, Department of Pediatrics, University of Kentucky, Lexington, KY, United States; ^2^Department of Pediatrics, Johns Hopkins All Children's Hospital in Florida, St. Petersburg, FL, United States; ^3^Department of Biostatistics, College of Public Health, University of Kentucky, Lexington, KY, United States

**Keywords:** chronic lung disease, BPD definition, premature, infants, bronchopulmonary dysplasia

## Abstract

**Objectives:** To compare the Shennan's and the consensus definition of Bronchopulmonary Dysplasia (BPD) from the National Institutes of Health (NIH) workshop and analyze specific risk factors associated with each definition.

**Study design:** Retrospective analysis of records of 274 infants admitted to a level IV intensive care unit. Infants were classified as having BPD or no BPD by both definitions. Differences in incidence and risk factors were analyzed. Statistical methods included descriptive statistics, comparative tests, and marginal logistic regression modeling.

**Results:** The estimated difference in prevalence was 32% [95% CI: (26%, 37%), (*p* < 0.0001)] between both criteria. The prevalence of BPD was 80% higher based on the NIH criteria [RR = 1.80; 95% CI: (1.58, 2.06)]. Infants with no BPD by the Shennan definition were breathing room air with or without positive or continuous pressure support and were most likely to be discharged home on oxygen [OR = 4.47, 95% CI: (1.20, 16.61), *p* = 0.03]. Gestational age, birth weight, and 1-min Apgar score predicted BPD by both definitions. Chorioamnionitis increased the risk of BPD by the Shennan definition but was associated with lower risk by the NIH criteria. IUGR was associated with BPD by the Shennan definition and with severe BPD by the NIH criteria.

**Conclusion:** Compared to the Shennan's definition, the NIH consensus identified 80% more infants with BPD and is a better predictor of oxygen requirement at discharge. Until a new better criteria is develop, the NIH consensus definition should be used across centers.

## Introduction

Bronchopulmonary dysplasia (BPD) is a term coined in 1967 to describe the clinical, pathologic and radiographic features of preterm infants that require prolonged mechanical ventilation and oxygen support (1). Preterm infants initially developed hyaline membrane disease and, despite the high mortality rate, survived, but with severe mucosal, alveolar, and vascular changes due to the prolonged exposure to high ventilator pressures and oxygen (1). As survival improved and new advances in neonatal care were introduced, infants developed a different BPD, sometimes described as a “new BPD,” which was characterized by an arrest in alveolar development, less fibrosis, and a more uniform inflammation (2, 3). In spite of the advances in the identification of factors that are related to the development of BPD (4–10) and its prevention (11, 12), BPD continues to be the most prevalent sequelae among survivors following preterm birth (13, 14).

Defining BPD has been a topic of debate ever since the initial criteria was proposed (3, 15–17). The original definition included infants with oxygen requirement for 28 days with associated radiographic changes (3, 17). Shennan et al. found that oxygen requirement for the first 28 days provided a poor positive predictive value for abnormal pulmonary findings as the gestational age decreases. However, oxygen need at 36 weeks Post-Menstrual Age (PMA) predicted abnormal pulmonary outcomes better (3, 18). Therefore, oxygen requirement at 36 weeks PMA became the criteria for BPD definition in infants born with birth weight (BW) < 1,500 g (18). This is the definition adopted by the Vermont-Oxford Network (19). However, the continued use of oxygen for the first 28 days is influenced by variation in clinical practices or protocols established at different centers (3, 14).

In lieu of the original definition, a new definition was proposed in 2001 as summarized by Jobe and Bancalari (3) from the discussions in a workshop organized by the National Institutes of Health (NIH). The definition of BPD differed according to gestational age (GA). For those born at GA < 32 weeks, BPD referred to requirement of oxygen support (>21%) for at least 28 days and a subsequent assessment at 36 weeks PMA or discharge, whichever comes first. In those born with GA > 32 weeks, BPD referred to the requirement of supplemental oxygen < 21% for at least 28 days and a subsequent assessment at 56 days post-natal age or discharge, whichever comes first. At the time of this assessment, those infants with no oxygen requirement were classified as having mild BPD. Moderate BPD was diagnosed in those requiring < 30% oxygen and severe BPD in those with a need for positive pressure ventilation/continuous positive pressure (PPV/CPAP) and/or oxygen requirement ≥30% (3). With the use of the new severity-based definition, 68% of infants admitted to centers from the Neonatal Research Network were diagnosed to have BPD (27% mild, 23% moderate and 18% severe) during the years 2003–2007 (17). This definition has since been adopted by the Eunice Kennedy Shriver National Institute of Child Health and Development Neonatal Research Network (3).

Using one definition or another can cause differences in the incidence of BPD. In 2005, a study by Ehrenkranz et al. found that 44% of infants were diagnosed with BPD as defined by oxygen use at 36 weeks PMA compared to 77% by using the NIH definition (20). These findings were confirmed by Poindexter et al. who found a 10% difference in the no BPD diagnosis between both definitions (21). Despite this, different centers across the country, as well as several multicenter trials and quality improvement initiatives, use either definition in characterizing infants with or without BPD (13, 20, 22–25). This study aimed to compare the BPD definitions by NIH and Shennan in an effort to understand how the differences between these two definitions would have an impact in clinical practice and follow up after discharge. As a secondary objective, we evaluated the factors that would predict BPD by each definition.

## Patients and Methods

This retrospective study was conducted with a cohort of premature infants admitted to a level IV Neonatal Intensive Care Unit (NICU) during 2013, 2014, and 2015. This study was approved by the Institutional Review Board. Electronic medical records of the study infants were reviewed and pertinent variables were entered into the password protected data base (REDCap software, Version 6.13.2 @ 2016 Vanderbilt University, Nashville TN) (26). Inclusion criteria were admission to the NICU, GA ≤ 30 weeks, and survival up to 36 weeks PMA. Exclusion criteria included GA > 30 weeks, presence of significant congenital anomalies, or death before 36 weeks PMA.

Prenatal factors, such as use of antenatal steroids, maternal chorioamnionitis, intrauterine growth restriction, preeclampsia and prolonged rupture of membranes were noted. Other variables recorded were BW, GA, inborn/outborn status, gender, Apgar scores at 1 and 5 min, mode of delivery, surfactant administration and length of stay in days. Infants who survived at 36 weeks PMA were categorized as BPD or No BPD based on the Shennan and NIH criteria. The BPD outcome of interest was need for oxygen at discharge or death after 36 weeks PMA, whichever comes first.

### Statistical Analysis

Descriptive statistics included determination of either means ± standard deviations or medians with interquartile ranges (IQR) for continuous variables and frequencies and percentages for categorical variables. McNemar's test was used to compare incidences between the two different BPD criteria, and generalized estimating equations (GEE) (27) were used to estimate the difference and ratio of these incidences. Comparisons of BPD and No BPD classifications for each definition separately were conducted utilizing two-sample *t*-tests, Wilcoxon tests, and either chi-square or Fisher's exact tests. Comparisons of Shennan vs. NIH criteria with respect to the impacts of potential predictors on the odds of BPD were made using GEE and marginal logistic regression modeling. Furthermore, with respect to oxygen requirement at 28 days, we calculated the C statistic, an estimate for the area under the receiver-operating characteristic curve and thus the predictive accuracy of this variable, and the optimal cutoff value based on the minimum Euclidean distance and maximum sum of sensitivity and specificity. Additionally, we determined the sensitivities, specificities, positive predictive values, negative predictive values, and the proportion correctly classified based on these optimal cutoffs. Tests were two-sided, with statistical significance defined as *p* < 0.05. Analyses were conducted in SAS version 9.4 (SAS Institute, Cary, N.C.).

## Results

A total of 247 infants fulfilled our inclusion criteria. Of these, 38 infants were evaluated at discharge and 209 at 36 weeks GA. Mean GA was 27.0 ± 1.7 weeks, mean BW was 975 ± 268 g, 58% were males and 29% of the infants were transferred to our facility. Sixty-eight percent and 12% of the infants were born after a full course and partial course of antenatal steroids, respectively. Delivery was by cesarean section in 72% of the infants. The median (range) Apgar scores were 4 (2, 6) and 6 (4, 8) at 1 and 5 min, respectively. Surfactant in the delivery room was given to 88% of the infants; of these, 31% received a second dose and 7% received a third dose. The average length of stay was 87 ± 43 days.

Regarding BPD classification in our cohort, 39% (97 infants) were found to require oxygen at 36 weeks and were classified to have BPD by the Shennan definition, compared to 71% (175 infants) using the NIH definition. Of those with BPD by NIH criteria, 17% (42), 4% (11), and 49% (122 infants) were classified as mild, moderate and severe BPD, respectively. Additionally, 53.9% of those with mild BPD by the NIH definition were classified as not having BPD using the Shennan definition. Table 1 depicts the classification of infants by both definitions. The difference in BPD incidence between criteria was significant (*p* < 0.0001). Specifically, the estimated difference in incidence was 32% [95% CI: (26%, 37%)], such that the incidence was estimated to be 80% higher based on the NIH criteria [RR = 1.80; 95% CI: (1.58, 2.06)].

**Table 1:** Number of subjects with BPD grouped by the NIH and the Shennan definitions.

	**NIH BPD** **negative**	**NIH BPD** **mild**	**NIH BPD** **moderate**	**NIH BPD** **severe**	**Total**
Sheenan BPD	0	0	9	88	97
Shennan BPD negative	72	42	2	34	150
Total	72	42	11	122	247

Of those classified as no BPD by the Shennan's definition, 78 infants were identified as having BPD by the NIH definition. Of these, 42, 2, and 34 infants were classified as mild, moderate and severe BPD, respectively. Further analysis showed that 34/78 (43.6%) were receiving some form of positive pressure ventilation (41% on CPAP, 1.3% on NIPPV, and 1.3% on a mechanical ventilator) and the remaining 2/78 (2.6%) were on nasal cannula.

In order to evaluate how maternal, perinatal, and infant factors are predictive of BPD, we analyzed their association with each BPD definition (Table 2). An increase of 1 week in GA [Shennan-OR = 0.56, 95% CI: (0.46, 0.69), *p* < 0.0001; NIH-OR = 0.44, 95% CI: (0.34, 0.56), *p* < 0.0001], an increase of 1 in Apgar score at 1-min [Shennan-OR = 0.88, 95% CI: (0.79, 0.99), *p* = 0.04; NIH-OR = 0.78, 95% CI: (0.69, 0.89), *p* = 0.0001], and an increase of 100 gm in BW [Shennan-OR = 0.69, 95% CI: (0.60, 0.78), *p* < 0.0001; NIH-OR = 0.63, 95% CI: (0.55, 0.71), *p* < 0.0001] were associated with significant decreases in odds of BPD by either definition; however, the decrease is significantly greater when using the NIH definition (*p* ≤ 0.04).

**Table 2:** Patient demographics per BPD definition.

	**Shennan**		**NIH**			
	**BPD** ***N* = 97**	**No BPD** ***N* = 150**	**Severe** ***N* = 122**	**Moderate** ***N* = 11**	**Mild** ***N* = 42**	**No BPD** ***N* = 72**
Gestational age (weeks)^a^	26.2 ± 1.6	27.6 ± 1.4	26.4 ± 1.6	26.2 ± 1.3	26.9 ± 1.4	28.2 ± 1.2
Birth weight (grams)^a^	842 ± 228	1,061 ± 257	871 ± 232	893 ± 202	956 ± 231	1,173 ± 246
Male^a^	65 (67%)	79 (53%)	78 (64%)	10 (91%)	23 (55%)	33 (46%)
Female^a^	32 (33%)	71 (47%)	44 (36%)	1 (9%)	19 (45%)	39 (54%)
**ANTENATAL STEROIDS**						
Complete	65 (69%)	95 (67%)	77 (65%)	7 (70%)	30 (77%)	46 (69%)
Partial	10 (11%)	19 (13%)	28 (24%)	0 (0%)	4 (10%)	11 (16%)
None	19 (20%)	27 (19%)	14 (12%)	3 (30%)	5 (13%)	10 (15%)
Intrauterine growth restriction^c^	13 (14%)	8 (5%)	16 (13%)	1 (9%)	2 (5%)	2 (3%)
Preeclampsia	19 (20%)	30 (20%)	30 (25%)	0 (0%)	8 (19%)	11 (15%)
Prolonged premature rupture of membranes	34 (35%)	41 (27%)	37 (30%)	6 (55%)	9 (21%)	23 (32%)
Chorioamnionitis^b^	11 (11%)	13 (9%)	13 (11%)	0 (0%)	2 (5%)	9 (13%)
Cesarean section	70 (72%)	108 (72%)	94 (77%)	8 (73%)	29 (69%)	47 (66%)
Vaginal delivery	27 (28%)	41(28%)	28 (23%)	3 (27%)	13 (31%)	24 (34%)
Inborn	65 (67%)	108 (72%)	81 (66%)	8 (77%)	31 (74%)	53 (74%)
Outborn	32 (33%)	42 (28%)	41 (34%)	3 (23%)	11 (26%)	19 (26%)
Apgar score 1 min^b^	3 (2, 5)	4 (2, 6)	3 (1, 5)	4 (3, 7)	5 (2, 6)	5 (3, 7)
Apgar score 5 min	6 (4, 7)	7 (5, 8)	6 (4, 7)	5 (3, 8)	7 (5, 8)	7 (6, 8)
**SURFACTANT**^**a**^						
1st dose at delivery	93 (96%)	124 (83%)	119 (98%)	9 (82%)	36 (86%)	53 (74%)
2nd dose	46 (49%)	29 (20%)	56 (47%)	2 (18%)	8 (20%)	9 (13%)
3rd dose	13 (13%)	4 (3%)	14 (11%)	1 (9%)	2 (5%)	0 (0%)
28 days oxygen use^a^	95 (99%)	79 (53%)	116 (96%)	10 (91%)	40 (95%)	8 (11%)
Home on oxygen or death before discharge^a^	87 (90%)	17 (11%)	90 (74%)	9 (82%)	3 (7%)	2 (3%)
Length of stay (days)^a^	99 (79, 115)	71 (57, 85)	94 (79, 108)	75 (65, 105)	74 (65, 88)	58 (49, 75)

*Mean ± SD, median (IQR)*.

a *p < 0.05 with respect to the comparison of BPD and No BPD based on the given condition*.

b *p < 0.05 for the comparison of Shennan vs. NIH with respect to the impact the given variable has on the odds of BPD*.

c *p = 0.04 for the comparison of BPD vs. No BPD and p = 0.02 for severe BPD vs. No BPD by NIH*.

Furthermore, having chorioamnionitis was estimated to increase the odds of BPD based on the Shennan's definition [OR = 1.36, 95% CI: (0.58, 3.19), *p* = 0.47], but decreased the odds based on the NIH definition [OR = 0.66, 95% CI: (0.28, 1.58), *p* = 0.35]. These disparate results between definitions (*p* = 0.04) correlates with the controversy that chorioamnionitis is a risk factor for BPD for some authors but not all (10, 17). Of note, IUGR was significantly associated with BPD by the Shennan definition and with severe BPD by NIH criteria.

Of all the factors that were associated with BPD, oxygen use at 28 days post-natal age was the best predictor for the development of BPD by either definition. Oxygen use at 28 days had a comparable sensitivity, 95 and 99%, respectively, as predictor of BPD by NIH and the Shennan criteria. However, the use of oxygen at 28 days had a better specificity, positive predictive value and higher proportion of correctly classified when defining BPD using NIH criteria. Oxygen use at 28 days had the best negative predictive value (99%) for BPD by Shennan's definition. Further, we estimated that the optimal cutoff for oxygen requirement at 28 days were FiO_2_ of 0.22 and 0.24, respectively, as predictor of BPD by NIH and Shennan's criteria (Table 3).

**Table 3:** Oxygen use at 28 days of life.

**Definition**	**Sn**	**Sp**	**PPV**	**NPV**	**PCC**	**C statistic**	
**ANY OXYGEN USE**							
NIH	0.95	0.89	0.95	0.89	0.93	0.92	
VON	0.99	0.47	0.55	0.99	0.67	0.73	
**Definition**	**Cutoff**	**Sn**	**Sp**	**PPV**	**NPV**	**PCC**	**C statistic**
**LOWEST OXYGEN LEVEL**							
NIH	22	0.759	1.000	1.000	0.612	0.825	0.880
VON	24	0.879	0.783	0.727	0.908	0.821	0.885

We also assessed the need for oxygen at discharge or death before discharge after 36 weeks PMA. Of those with no BPD, 11 and 3% of the infants by Shennan and NIH criteria, respectively, were discharged home on oxygen or died before discharge. Infants who were classified as having no BPD by Shennan were more likely to be discharged home on oxygen compared to those classified as not having BPD by the NIH definition [OR = 4.47, 95% CI: (1.20, 16.61), *p* = 0.03].

## Discussion

The incidence of BPD using the NIH criteria is 80% higher when compared to the Shennan's definition with an estimated difference of 32%. This difference is consistent with other publications (20, 21). Associated with either definition are the risk factors: BW, GA, male sex, IUGR, low Apgar scores, intubation in the delivery room, and additional surfactant doses. Infants classified as not having BPD by the Shennan criteria are more likely to go home on oxygen compared to those classified as no BPD by the NIH criteria.

Throughout the years, the characteristics and treatment of infants who developed BPD have remarkably changed since it was initially described by Northway (1). The widespread uses of antenatal steroids and surfactant administration have reduced the risk of BPD in the at-risk population (28). Nevertheless, BPD continues to be a major cause of morbidity and mortality in the extremely low birth weight (ELBW) infants (24, 28, 29). Because studies in the past several years have drawn attention to the associated unfavorable pulmonary or functional outcomes related to BPD, prediction models or scoring systems have been proposed to identify those at risk (4, 6–9, 15, 30)^1^. Most prediction models have included similar perinatal risk factors that we noted in our study (8, 31). In our cohort as well as in other studies, oxygen use at 28 days post-natal age was the best predictor of BPD by either definition (18), more specifically any oxygen use when using the NIH criteria. This observation has the potential to be included when developing BPD predictor models (18, 32).

The definition of BPD in a preterm infant carries an important influence in predicting future health issues or interventions (24, 29, 33–36). By Shennan's criteria 50% of infants receiving oxygen at 36 weeks PMA were destined to have abnormal pulmonary outcomes as measured by re-hospitalization secondary to respiratory infection or disease like asthma or the need for pulmonary medications, such as bronchodilators or steroids (18). Similarly, by using the NIH criteria, Ehrenkranz et al. reported an increasing incidence of adverse pulmonary outcome as BPD becomes more severe (20).

The prevalence of BPD has been used as an index of quality of care when comparing different centers (23). Specifically, it is used as an estimate of center-specific therapeutic strategy-effectiveness as well as a gauge of when management protocols need to be updated (37–39). Of concern is that in our cohort, 80% more infants were identified as having BPD by NIH compared to Shennan's definition which fails to identify infants on respiratory support at 36 weeks GA as having BPD. Contrary to expectation, the inclusion of need for respiratory support in the definition has not been widely accepted across major centers (23, 32, 40). By not correctly classifying these infants, it becomes harder for clinicians and researchers to evaluate the real impact of BPD on long term pulmonary outcomes (11, 25, 37). Those with no BPD by the Shennan definition were four times more likely to go home on oxygen compared to those with no BPD by NIH criteria. A measure that is highly predictive at 36 weeks PMA of the need for oxygen on discharge is relevant to assist families, even prior to discharge, to access resources and medical services that will be needed for weeks or months after NICU hospitalization (24, 34, 36, 39).

Bronchopulmonary dysplasia has also been associated with changes in the pulmonary vasculature of affected infants like, decreased in the cross-sectional vascular bed, increased pulmonary vascular tone and reactivity (41). Therefore, infants that are diagnosed with BPD have an increased risk of developing pulmonary artery hypertension (PH) which adds significantly to the morbidity and mortality of these infants (42). The presence of IUGR and SGA have been identify as an additional risk factor for the development of PH (31, 41, 43). However, the evaluation of PH in infants with BPD is highly variable as well as its management (31, 42). Even though none of the current definitions assist with stratification of risk for BPD/PH, a standardize use of a definition would aid in the identification of infants at risk for PH.

A third definition of BPD, the “physiologic definition,” was proposed in 2003 (44). Infants that required positive pressure and oxygen ≥0.3 at 36 weeks PMA were classified as BPD, those requiring FiO_2_ < 0.3 required an oxygen reduction test for up to 2 weeks based on oxygen saturations (44). However, this definition has not gained popularity. Compared to the definition of oxygen requirements at 36 weeks PMA, the “physiologic definition” took in consideration the use of positive pressure and showed a 10% reduction in the incidence of BPD with less variability among centers (45).

In summary, BPD incidence differs depending upon the definition used. Furthermore, if the definition is based on oxygen requirement alone, being in room air at 36 weeks is not necessarily a predictor of not requiring oxygen at discharge. Lacking the capability into determination of alveolar and pulmonary vascular pathology, we strongly urge for a consensus in defining BPD from a clinical perspective, if BPD is to serve as one of clinical outcome measures across centers. Indeed, a sound recommendation on which definition to use will standardized the incidence of BPD as alluded to by Bancalari et al. (25). Also the definition is to be such that it will not only identify those infants with BPD but will also be a predictor of the pulmonary care needs at discharge and follow-up.

## Author Contributions

EG had primary responsibility for protocol development, outcome assessment, preliminary data analysis, and writing the manuscript. VC and AS participated in the development of the protocol, patient screening, enrollment, and writing of the manuscript. PW participated in the protocol design, was responsible for all the statistical analysis and contributed to the writing of the manuscript. HB supervised the design and execution of the study, performed the final data analyses and contributed to the writing of the manuscript.

### Conflict of Interest Statement

The authors declare that the research was conducted in the absence of any commercial or financial relationships that could be construed as a potential conflict of interest.

## Footnotes

^1^Palta 1998 Evaluation of Criteria for CLD.pdf.
